# Analysis and prediction of air quality in Nanjing from autumn 2018 to summer 2019 using PCR–SVR–ARMA combined model

**DOI:** 10.1038/s41598-020-79462-0

**Published:** 2021-01-11

**Authors:** Bing Liu, Yueqiang Jin, Chaoyang Li

**Affiliations:** 1 Public Foundational Courses Department, Nanjing Vocational University of Industry Technology, Nanjing, 210023 China; 2 College of Management, Henan University of Technology, Zhengzhou, 450001 China

**Keywords:** Climate sciences, Environmental sciences

## Abstract

In order to correct the monitoring data of the miniature air quality detector, an air quality prediction model fusing Principal Component Regression (PCR), Support Vector Regression (SVR) machine, and Autoregressive Moving Average (ARMA) model was proposed to improve the prediction accuracy of the six types of pollutants in the air. First, the main information of factors affecting air quality is extracted by principal component analysis, and then principal component regression is used to give the predicted values of six types of pollutants. Second, the support vector regression machine is used to regress the predicted value of principal component regression and various influencing factors. Finally, the autoregressive moving average model is used to correct the residual items, and finally the predicted values of six types of pollutants are obtained. The experimental results showed that the proposed combination prediction model of PCR–SVR–ARMA had a better prediction effect than the artificial neural network, the standard support vector regression machine, the principal component regression, and PCR–SVR method. The Root Mean Square Error (RMSE), Mean Absolute Error (MAE), and relative Mean Absolute Percent Error (MAPE) are used as evaluation indicators to evaluate the PCR–SVR–ARMA model. This model can increase the accuracy of self-built points by 72.6% to 93.2%, and the model has excellent prediction effects in the training set and detection set, indicating that the model has good generalization ability. This model can play an active role scientific arrangement and promotion of miniature air quality detectors and grid-based monitoring of the concentration of various pollutants.

## Introduction

Around the world, about 3 million people die every year due to air quality problems. Many studies have shown that atmospheric pollutants have a certain correlation with lung cancer and cardiovascular disease^1–3^. Chinese cities, like many other cities in the world, are facing challenges in their fight against air pollution. Although many cities have made many efforts to prevent air pollution, they still do not meet the requirements of air quality regulations (GB3095-2012). For this reason, relevant national departments need to monitor the concentration of major pollutants in the atmosphere in real-time, so as to grasp air quality in time and take corresponding measures against pollution sources.

### Low-cost air quality platforms

There are three main purposes of air quality monitoring: First, through regular or continuous monitoring of the main pollutants in the air environment, to determine whether the air quality meets the national air quality standards, and to provide data for the preparation of air environment quality evaluation reports. Second, it provides a basis for studying the laws and development trends of air quality, and for predicting and forecasting air pollution. Third, provide basic information and basis for government departments to implement relevant environmental protection laws and regulations, carry out environmental quality management, environmental scientific research, and revise atmospheric environmental quality standards. The air monitoring items mainly include PM2.5, PM10, CO, NO_2_, SO_2_, and O_3_ (''two dusts and four gases"). Air monitoring is the basis for air quality control and reasonable evaluation of air quality.

Many large cities have some monitoring stations that monitor air quality. However, the cost of installing and maintaining reference monitoring stations (national control points) is very high, so the distribution of monitoring stations is relatively sparse and can only monitor air quality in a few places. In addition, the release of data from national control points has a long lag, and it cannot provide real-time air quality monitoring and forecasting. The miniature air quality detector (self-built point) developed by some enterprises is not only low-cost, but also allows real-time grid monitoring of the air quality in a certain area, and simultaneously monitors meteorological parameters such as temperature, humidity, wind speed, pressure, and precipitation in the area.

The miniature air quality detector has the advantages of low cost, light weight and convenient installation. It generally uses solar panels, accumulators and AC power supply. When the external current is cut off, the accumulator can be used for power supply. Even if it is powered by the accumulator, it can still work stably within 48 h. Monitoring air quality with a miniature air quality detector has now become a popular trend in the world, and new equipment is constantly being updated. The miniature air quality detectors have been extensively checked and calibrated in laboratory. However, the results change dramatically when the devices are deployed in urban sites^4,5^. Many miniature air quality detectors use electrochemical sensors, which work by reacting with the gas to be measured and generating an electrical signal proportional to the gas concentration. Electrochemical sensors will produce zero drift and range drift after using for a certain period of time. Changes in the concentration of unconventional gaseous pollutants and weather factors will also interfere with the sensor's measurement, which causing errors in the measurement data. Therefore, it is particularly important to use the national control point data to calibrate the corresponding self-built point data.

### Introduction to air quality prediction model

Many scholars have conducted research on air quality prediction models. In general, air quality forecasting models mainly include statistical models based on machine learning and mechanism models based on atmospheric chemical analysis. The mechanism model of atmospheric chemical analysis is based on human scientific understanding of atmospheric physics and chemical processes. It uses mathematical methods combined with meteorological principles to simulate the physical and chemical processes of pollutants, simulate the processes of pollutants transport, reaction, and removal in the atmosphere, and uses the generated gridded data of pollutants to achieve air quality monitoring^6^. The mechanism model has high accuracy in weather forecasting, but it is not accurate in predicting the concentration of pollutants. Statistical models based on machine learning use statistical methods to analyze and model the collected pollutant data, and use mathematical algorithms to mine the internal relationships between variables from the data set.

Many literatures have used different statistical methods to study air quality models. Linear regression analysis models are usually used to discuss air quality issues^7–9^. The advantages of this model are simple calculation, easy interpretation of regression coefficients, and unique output results, but it is not suitable for dealing with nonlinear problems. The Markov model is often used to predict the concentration of pollutants in the air^10,11^. This method has a good effect on the state of the process, but it is not suitable for medium and long-term prediction of the system. Some scholars use random forest algorithm^12–14^ to build air quality prediction models, but random forests have been proven to overfit in some noisy classification or regression problems. There are also some literatures using artificial neural networks^15–17^ to establish air quality prediction models. The artificial neural network turns the characteristics of all problems into numbers and turns all reasoning into numerical calculations, and is unable to explain its own reasoning process and reasoning basis.

Support Vector Regression (SVR) machine is a machine learning method based on statistical learning theory that minimizes structural risk. It can not only overcome the problems of traditional prediction methods in small sample and high-dimensional application scenarios, but also has better generalization performance and nonlinear fitting capabilities. Therefore, the support vector regression machine has been well applied in the air quality prediction model^18,19^. The support vector regression machine was used by Ortiz-Garcia, E. G., et al. to predict the hourly O_3_ concentration in Madrid, and the prediction results were compared with the artificial neural network results^20^. A. Suarez Sanchez, et al. established an air quality regression model using support vector machine technology in the urban area of Aviles, Spain (Spain) based on experimental data of air pollutants from 2006 to 2008^21^. Sheng, Jiao, et al. used support vector regression to predict the dust concentration in the urban atmospheric environment^22^.

There are many factors that affect air quality, and the concentration of pollutants is cross-influenced by many external factors. If various factors are directly introduced into the air quality model, the information cannot be fully used due to the cross-influence between the various factors. In order to solve this problem, this paper first uses Principal Component Regression (PCR) to extract the main information of each factor, and then uses the support vector regression machine to regress the predicted value of the principal component regression and each factor, making full use of the information contained in each factor. Many air quality models do not extract valuable information from the error term. We use the Autoregressive Moving Average (ARMA) model to extract valuable information from the residuals, and modify the model prediction values to improve the prediction accuracy. The PCR–SVR–ARMA model is not only highly interpretable, but also has a good predictive effect, and since the calculation of SVR is basically not affected by the dimension of the sample space, the complexity of the model is not significantly improved. The empirical results show that the prediction results of this model can predict the concentration of pollutants very well, and further provide a basis for data correction of the miniature air quality detector.

## Material and methods

### Data source and preprocessing

Due to the sparse deployment of national control points, it is difficult to conduct grid-based monitoring of the concentration of pollutants in the air. Therefore, miniature air quality detectors can be promoted to monitor air quality. Because the electrochemical sensor in the miniature air quality detector receives the influence of many internal and external factors, it will cause the measurement to be biased, so we use the statistical model to correct.

This article selects 2019 Chinese college students' mathematical modeling D problem data. It provides data on two sets of collocated monitoring points in Nanjing. One set of data is the hourly data of national control points from November 14, 2018 to June 11, 2019, and the other set of data is self-built point data corresponding to the national control points. There are a total of 4200 sets of data provided by the national control point. Each set of data contains the concentration of six types of pollutants, and the interval between them is one hour. There are a total of 234,717 sets of data provided by self-built sites, each set of data includes six types of pollutant concentrations and five types of meteorological parameters at the time. corresponding to the national control point, and its time interval does not exceed 5 min.

Before conducting exploratory analysis on the data of national control points and self-built points, the data is pre-processed. First, delete the data that cannot correspond to the time of the self-built point and the national control point. We consider values greater than 3 times the average of the left and right adjacent values as outliers and delete them. The occurrence of outliers may be caused by accidental emissions or errors in measuring instruments. Second, the various data within each hour of the self-built point are classified and aggregated and averaged to correspond to the hourly data of the national control point. After data preprocessing, a total of 4,135 sets of data were obtained as research objects. Table 1 shows the range, mean, and standard deviation of each variable.

**Table 1:** Descriptive statistics of air quality variables from data from national control points and self-built points.

Input variable	Ranges	Mean	Standard deviation
PM2.5/(μg/m^3^)	1–216.883	64.127	37.328
PM10/(μg/m^3^)	2–443.25	102.391	65.267
CO/(μg/m^3^)	0.05–3.895	0.863	0.452
NO_2_/(μg/m^3^)	0.947–157.136	45.209	28.403
SO_2_/(μg/m^3^)	1–651.3	19.397	18.723
O_3_/(μg/m^3^)	0.579–259	61.586	40.941
Wind speed/(m/s)	0.133–2.387	0.7	0.346
Pressure /(Pa)	996.871–1039.8	1018.8	8.889
Precipitation /( mm/m^2^)	0–312.1	132.084	87.004
Temperature /(℃)	− 3.882 to 37.944	11.882	8.603
Humidity /( rh%)	10.667–100	68.903	21.931

### Data exploratory analysis

The establishment of statistical models usually starts with exploratory analysis of the data^23^. Based on the data of national control points, this paper analyzes the concentration data of six types of pollutants measured by self-built points. First, because the self-built point data is given at 5-min intervals, it cannot correspond to the 1-h interval data of the national control point, so we average the hourly data of the self-built point to correspond to the national control point data, and get a total of 4135 sets of corresponding data. Second, in order to more intuitively reflect the difference between the national control point and the automatic control point data, this paper first calculates the daily average of the pollutant concentration data and then conducts a comparative analysis. Because the six pollutants concentration discussion methods are similar, we only discuss the NO_2_ concentration in detail.

**Figure 1:**
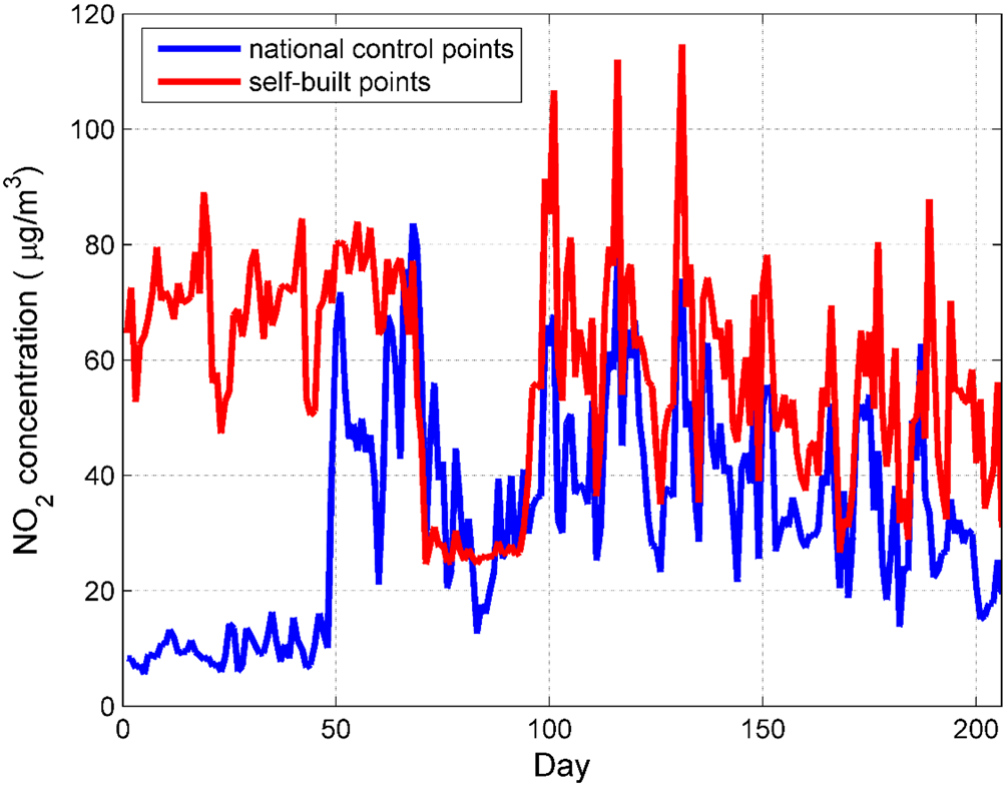
Comparison of daily average NO_2_ concentration data between national control points and self-built points. Figures are generated using Matlab (Version R2016a, https://www.mat- hworks.com/) [Software].

**Figure 2:**
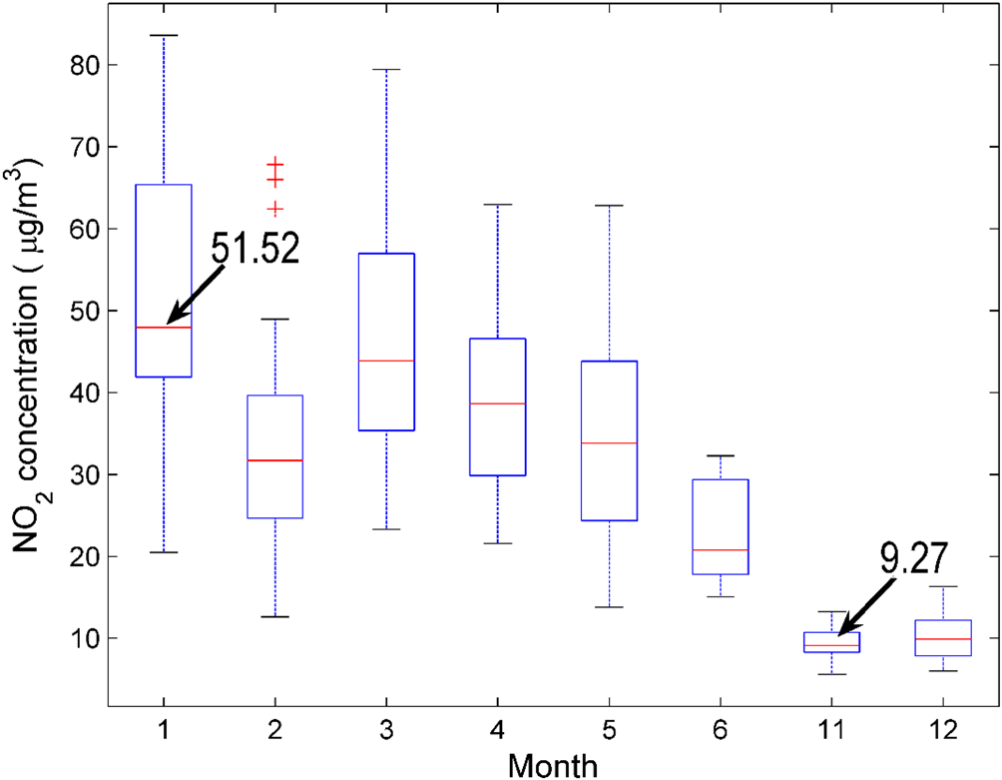
Compare the concentration of NO_2_ in national control points monthly. Note that there is no data from July to October.

It can be seen from Fig. 1 that the measurement data of the NO_2_ concentration national control point and the self-built point are generally consistent in the changing trend, but there are errors that cannot be ignored between the two. The two errors in the previous stage are obviously larger, which may be caused by the season or the zero drift of the measuring instrument. When the concentration of NO_2_ is low, the large error of the self-built point measurement data indicates that the miniature air quality detector has certain shortcomings. We are most concerned about the prediction accuracy when the NO_2_ concentration is high, so this defect is not too serious. Since the NO_2_ concentration fluctuates significantly over time, we draw a box plot of NO_2_ concentration changes with months^10^ as shown in Fig. 2.

From the box plot of NO_2_ concentration, it can be seen that the average NO_2_ concentration is highest in January and lowest in November (no data from July to October), the highest concentration is 51.52 μg/m^3^, and the lowest concentration is 9.27 μg/m^3^. There are obvious differences in NO_2_ concentration in different months, indicating that the NO_2_ concentration in the air presents obvious seasonal characteristics. Man-made emissions are the main reason for this situation. In winter, heating has led to an increase in energy use, forming an annual peak of nitrogen dioxide, increasing the difficulty of preventing and controlling heavy haze pollution. The humidity increases in summer and autumn, and the diffusion conditions are better, so the nitrogen dioxide content decreases.

### Correlation analysis

**Figure 3:**
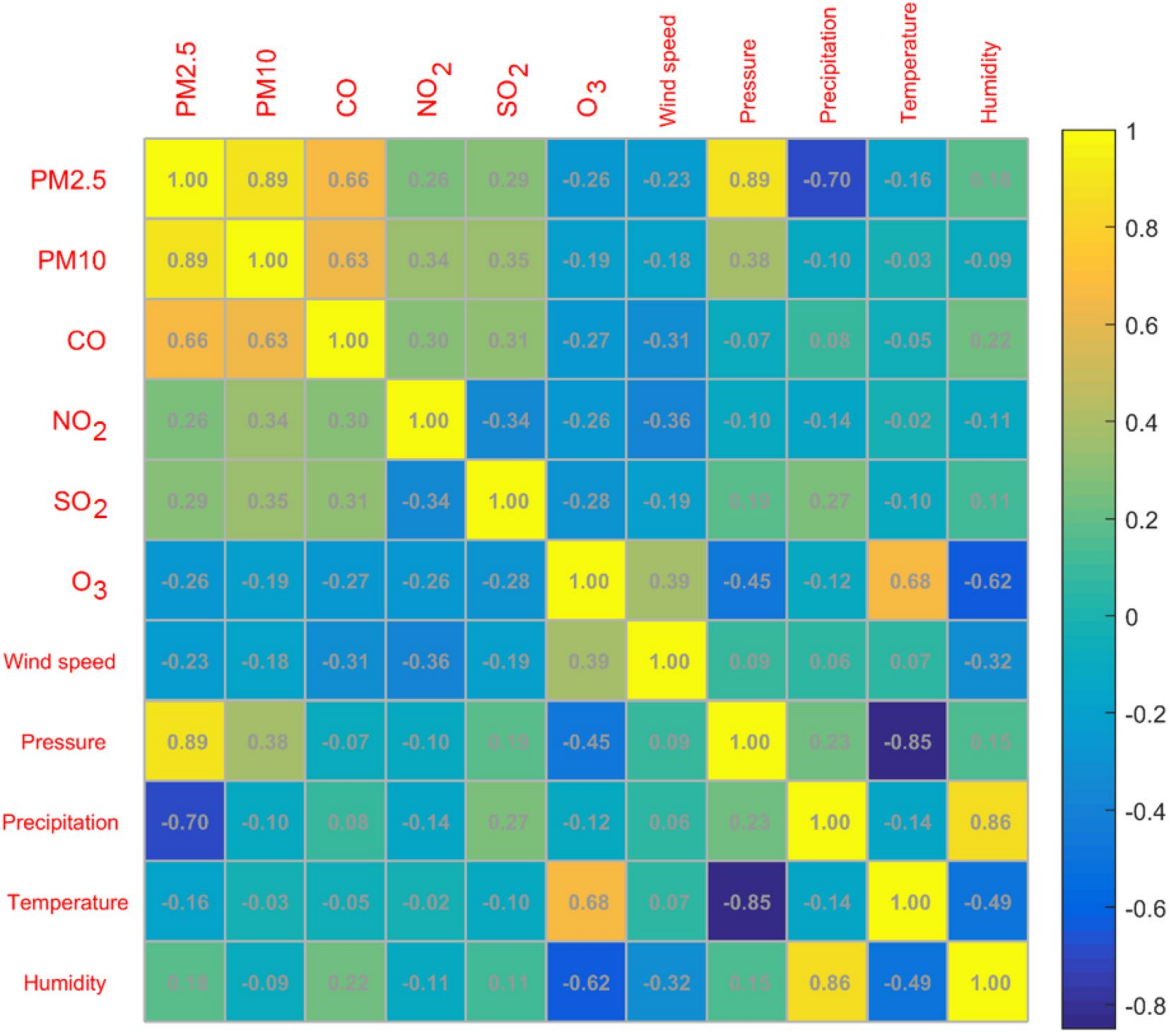
Pearson correlation coefficient matrix color block diagram between the concentration of “two dust and four gases” and climate factors.

Humans pay more and more attention to air quality. There are many factors that affect air quality, and they affect each other^24^. In order to find the relationship between the concentration of the six pollutants and the five climatic factors, Eq. (1) is used to find the Pearson correlation coefficient between them, where ${x}_{i}$ and ${y}_{i}$ represent the concentration of six types of pollutants and the values of five climate factors. $\overline{x}$, $\overline{y}$ represent the average value of ${x}_{i}$ and ${y}_{i}$, the results are shown in Table 2. It can be seen that, except for NO_2_ concentration and temperature, all other variables have significant correlations with each other, indicating that the factors affecting the concentration of each pollutant are very complex. The correlation coefficient between PM2.5 concentration and PM10 concentration is 0.89, indicating a positive correlation between the two, and the correlation coefficient between temperature and air pressure is − 0.85, indicating a negative correlation between the two. The correlation coefficient matrix color block diagram can intuitively display the correlation coefficient value. Figure 3 is a matrix color block diagram between the concentration of six types of pollutants and five climatic factors, which visually shows the correlation coefficients between variables.

**Table 2:** Pearson linear correlation coefficients between six types of air pollutant concentrations and climate (Band * indicates significant correlation at a significant level of 0.05).

Variable	PM2.5	PM10	CO	NO_2_	SO_2_	O_3_	Wind speed	Pressure	Precipitation	Temperature	Humidity
PM2.5	1.00	0.89*	0.66*	0.26*	0.29*	− 0.26*	− 0.23*	0.89*	− 0.70*	− 0.16*	0.18*
PM10		1.00	0.63*	0.34*	0.35*	− 0.19*	− 0.18*	0.38*	− 0.10*	− 0.03*	− 0.09*
CO			1.00	0.30*	0.31*	− 0.27*	− 0.31*	− 0.07*	0.08*	− 0.05*	0.22*
NO_2_				1.00	− 0.34*	− 0.26*	− 0.36*	− 0.10*	− 0.14*	− 0.02	− 0.11*
SO_2_					1.00	− 0.28*	− 0.19*	0.19*	0.27*	− 0.10*	0.11*
O_3_						1.00	0.39*	− 0.45*	− 0.12*	0.68*	− 0.62*
Wind speed							1.00	0.09*	0.06*	0.07*	− 0.32*
Pressure								1.00	0.23*	− 0.85*	0.15*
Precipitation									1.00	− 0.14*	0.86*
Temperature										1.00	− 0.49*
Humidity											1.00

The correlation coefficient between PM2.5 concentration and PM10 concentration is as high as 0.89, indicating a high positive correlation between the two, and the correlation coefficient between temperature and air pressure is − 0.85, which indicates that the higher the temperature, the lower the pressure. Figure 3 is a matrix color block diagram between the concentration of "two dusts and four gases" and five climatic factors, which visually shows the correlation coefficients between the variables. The size of the matrix color block represents the absolute value of the correlation coefficient. As the color becomes lighter, the value of the correlation coefficient gradually increases. The value in the matrix color block diagram represents the Pearson correlation coefficient of the corresponding variable. And the lighter the color, the larger the correlation coefficient value.

1 $$\mathrm{r}=\frac{\sum_{i=1}^{n}({x}_{i}-\overline{x})({y}_{i}-\overline{y})}{\sqrt{{\sum_{i=1}^{n}({x}_{i}-\overline{x})}^{2}}\bullet \sqrt{{\sum_{i=1}^{n}({y}_{i}-\overline{y})}^{2}}}$$

## Establishment of sensor calibration model

### Introduction to basic principles

Principal component analysis was first proposed by Hotelling in 1933. Principal component analysis is a traditional statistical analysis method that uses the idea of dimensionality reduction to convert multiple indicators into several comprehensive indicators using orthogonal rotation transformation under the premise of losing little information. The comprehensive index generated by transformation is usually called the principal component, where each principal component is a linear combination of the original variables, and the principal components are not related to each other^25^. From a mathematical point of view, solving the principal components is actually the process of solving the eigenvalues and eigenvectors according to the covariance matrix of the data source. The linear combination of the covariance matrix and the original variables is the principal component.

2 $$\left\{ \begin{gathered} Y_{1} = \mu_{11} X_{1} + \mu_{12} X_{2} + \cdots + \mu_{1p} X_{p} \hfill \\ Y_{2} = \mu_{21} X_{1} + \mu_{22} X_{2} + \cdots + \mu_{2p} X_{p} \hfill \\ \quad \quad \quad \vdots \hfill \\ Y_{p} = \mu_{p1} X_{1} + \mu_{p2} X_{2} + \cdots + \mu_{pp} X_{p} \hfill \\ \end{gathered} \right.$$

From Eq. (2), we know that there are several original variables, and several principal components will be obtained. In actual work, we only select the first few principal components with the largest variance, so as to simplify the system structure and grasp the essence of the problem. According to the characteristic roots of the covariance matrix, the appropriate principal components can be selected. The concept of contribution rate is shown in Eq. (3), where $\lambda$ is the characteristic root of the covariance matrix; and $P_{k}$ is the contribution rate of the kth principal component. Generally, the sum of the contribution rates of the first m principal components (cumulative contribution rate) is greater than 85. The first m principal components u are the principal components obtained after principal component analysis, generally m < P, so as to achieve the purpose of reducing the dimensionality of the independent variables and simplifying the system^26,27^. Principal component regression analysis (PCR) is a regression analysis with principal components as independent variables. It is a commonly used method to solve the problem of multicollinearity.

3 $$P_{k} = \lambda_{k} /\sum\nolimits_{i = 1}^{p} {\lambda_{i} }$$

**Figure 4:**
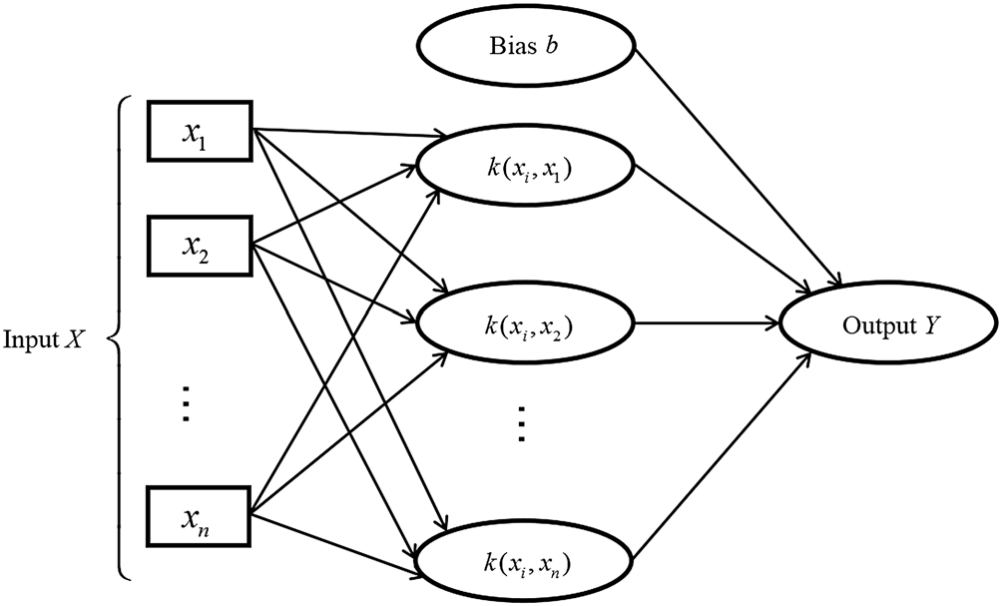
Support vector machine architecture.

In the 1990s, Vapnik et al. proposed the support vector machine theory based on small sample statistics. Its architecture is shown in Fig. 4. The basic principle of support vector machine is to use training error as the constraint condition to solve the problem, and the minimum confidence interval as the final goal of optimization. Its essence is to solve a convex programming or quadratic programming problem. The support vector machine first maps the nonlinear transformation problem to a high-dimensional space through the inner product kernel function, turning it into a linear problem to find the generalized classification surface or regression problem^28^.

For a given set of data $T = \{ (x_{1} ,y_{1} ), \ldots ,(x_{i} ,y_{i} )\} \subset R^{d} \times R,i = 1, \ldots ,n$, the regression problem we want to solve is simply to find the mapping relationship between $x_{i}$ and $y_{i}$.In Eq. (4) ,$[\omega ,\varphi (x)]$ corresponds to the inner product of $R^{d}$ space. $\varphi (x)$ is the kernel function, which maps the training sample data to the high-dimensional space $F$.

4 $$y = f(x) = [\omega ,\varphi (x)] + b,x \in R^{d} ;y,b \in R$$

Support vector machine regression theory expresses this kind of problem as searching for an optimal function $\{ f(x,\omega^{*} )\}$ in a set of functions $\{ f(x,\omega )\}$ so as to minimize the expected expected risk $R(\omega )$. Equation (5) is the expected risk $R(\omega )$, where $n$ is the sample size and $h$ is the $VC$ dimension.

5 $$R(\omega ) \le R_{emp} (\omega ) + \sqrt {\frac{h(\ln (2n/h) + 1) - \ln (\eta /4)}{n}}$$

6 $$\begin{aligned} W_{\max } & = - \frac{1}{2}\sum\limits_{i = 1}^{n} {\sum\limits_{j = 1}^{n} {(a_{i} - a_{i}^{*} )} } (a_{j} - a_{j}^{*} )k(x_{i} ,x_{j} ) - \varepsilon \sum\limits_{i = 1}^{n} {(a_{i} + a_{i}^{*} )} + \sum\limits_{i = 1}^{n} {y_{i} (a_{i} - a_{i}^{*} )} \\ & \quad s.{\kern 1pt} {\kern 1pt} {\kern 1pt} t.\;\left\{ \begin{gathered} \sum\limits_{i = 1}^{n} {(a_{i} - a_{i}^{*} ) = 0} \hfill \\ 0 \le a_{i} ,a_{i}^{*} \le C,i = 1,2, \cdots ,n \hfill \\ \end{gathered} \right. \\ \end{aligned}$$

The support vector machine converts the minimization of the expected risk $R(\omega )$ into seeking the optimal solution of Eq. (6), where ${a}_{i}$ is the corresponding Lagrange multiplier and ${a}_{i}^{*}$ is its optimal solution. $\varepsilon$ determines the flatness of the regression curve according to the insensitive loss function $L(y,f(x,a))$(Eq. (7)), and $0 < \varepsilon < 1$. When the error value between the actual result value $y$ at the point $x$ and the predicted value $f(x)$ does not exceed the predetermined $\varepsilon$, then the predicted value $f(x)$ at this point is considered to be lossless. In Eq. (6), $C$ is the penalty factor, which represents the penalty for the wrong classification of the sample.

7 $$L(y,f(x,a)) = L(\left| {y - f(x,a)} \right|_{\varepsilon } )$$

8 $$\begin{gathered} where:\quad \left| {y - f(x,a)} \right|_{{{\kern 1pt} \varepsilon }} = \left\{ \begin{gathered} 0{\kern 1pt} ,y - f(x,a) \le \varepsilon \hfill \\ \left| {y - f(x,a)} \right| - \varepsilon \,,{\text{others}} \hfill \\ \end{gathered} \right. \hfill \\ \hfill \\ \end{gathered}$$

From this, the optimal solution $a^{(*)} = (a_{1} ,a_{1}^{*} , \ldots ,a_{i} ,a_{i}^{*} )^{T}$ is obtained, and the regression function corresponding to the support vector machine is Eq. (9).

9 $$f(x) = \sum\limits_{i = 1}^{SV} {(a_{i} - a_{i}^{*} )} k(x_{i} ,x_{j} ) + b$$

In support vector machines, there are many types of non-linear kernel functions. Commonly used are polynomial function, radial basis function, sigmoid function (Eqs. (10)–(12)), etc. Considering the better performance of the radial basis function, this paper chooses it as the kernel function of the support vector machine.

10 $$k(x_{i} ,x_{j} ) = (\gamma x_{i}^{T} x_{j} + r)^{p} ,\gamma > 0$$

11 $$k(x_{i} ,x_{j} ) = \exp ( - \gamma \left\| {x_{i} - x_{j} } \right\|^{{{\kern 1pt} 2}} ){\kern 1pt} ,\gamma > 0$$

12 $$k(x_{i} ,x_{j} ) = \tanh (\gamma x_{i}^{T} x_{j} + r)$$

Establishing an air quality prediction model through SVR, although historical time series data can be used to train the SVR prediction model to obtain better prediction results, a series of error time series data is still obtained. The time series data composed of residuals has a certain degree of non-pure randomness and autocorrelation, and still hides valuable information that needs further mining and analysis. Therefore, it is necessary to use a suitable algorithm to construct a residual information extraction model to correct the SVR prediction results and further improve the prediction accuracy of the entire model.

Local simulation approximation, vector error correction, period extrapolation, Bayes vector method, autoregressive moving average model are often used to extract and correct residual information. Research shows that the ARMA time series method can not only better describe random time series data and further dig out valuable information, but also has the advantages of simple and efficient structure. Therefore, for the valuable information of the residual time series data that SVR failed to effectively extract, this research first checks the stationarity and pure randomness of the residual time series, and then extracts the valuable information of the residual through the ARMA model, and then revises the SVR model predictive value to improve forecast accuracy.

The expression of the ARMA model ARMA(p, q) is shown in Eq. (12), where p and q are the orders of the ARMA model, and u is the white noise time series. It satisfies $EU_{t} y_{t - 1} = 0$,$\varphi_{i} (i = 1,2, \ldots ,p)$ and $\psi_{i} (i = 1,2, \ldots ,q)$ are autoregressive parameters and moving average parameters respectively^29–31^.

**Figure 5:**
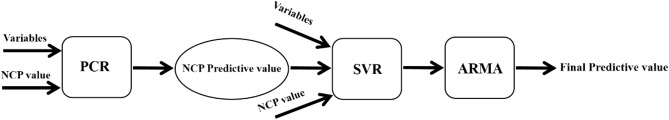
The flux diagram of the regression process.

In order to overcome the limitations of traditional statistical methods and effectively apply the powerful nonlinear regression capabilities of support vector machines, this study uses the PCR–SVR combined model to establish an air quality model. Principal component analysis is used to process the original data to obtain the corresponding principal components. Using multiple linear regression, the fitted values of the concentrations of various pollutants are obtained. The original independent variables and the fitted values of the principal component regression are used as input variables to establish a support vector machine regression model. The PCR–SVR model can not only retain most of the information of the original data, increase the interpretability of variables, but also increase the accuracy of the prediction model. The valuable information of PCR–SVR model residuals is extracted through the ARMA model, and finally the PCR–SVR–ARMA model is obtained. The process of building the model is shown in Fig. 5.

### Construction of PCR model

The relationship between the influencing factors of air quality is complicated, and the mutual influence between them can be seen from Table 2. The diagnosis of multicollinearity shows that the maximum variance inflation factor is 26.631, which is much greater than 10. Therefore, there is serious multicollinearity among influencing factors. Introducing all factors directly into the multiple linear regression model will increase the variance of the model and make the model very unstable, which will affect the application of the model. Eliminating some unimportant variables, increasing sample size, and biased estimation of regression coefficients are often used to solve multicollinearity. Principal component analysis is a commonly used method to eliminate multicollinearity. This article uses principal component analysis to eliminate multicollinearity. Calculate the contribution rate of each principal component through SPSS 20.0, as shown in Table 3.

**Table 3:** Principal component characteristic value and contribution rate of air pollutant concentration and climate factors.

Serial number	Principal component	Eigenvalues	Contribution rate %	Cumulative contribution rate %
1	1st principal component	3.213	29.208	29.208
2	2nd principal component	2.137	19.423	48.631
3	3rd principal component	1.485	13.504	62.135
4	4th principal component	1.138	10.345	72.48
5	5th principal component	1.021	9.283	81.763
6	6th principal component	0.647	5.878	87.642
7	7th principal component	0.591	5.373	93.015
8	8th principal component	0.409	3.72	96.735
9	9th principal component	0.281	2.557	99.292
10	10th principal component	0.057	0.52	99.812
11	11th principal component	0.021	0.188	100

**Figure 6:**
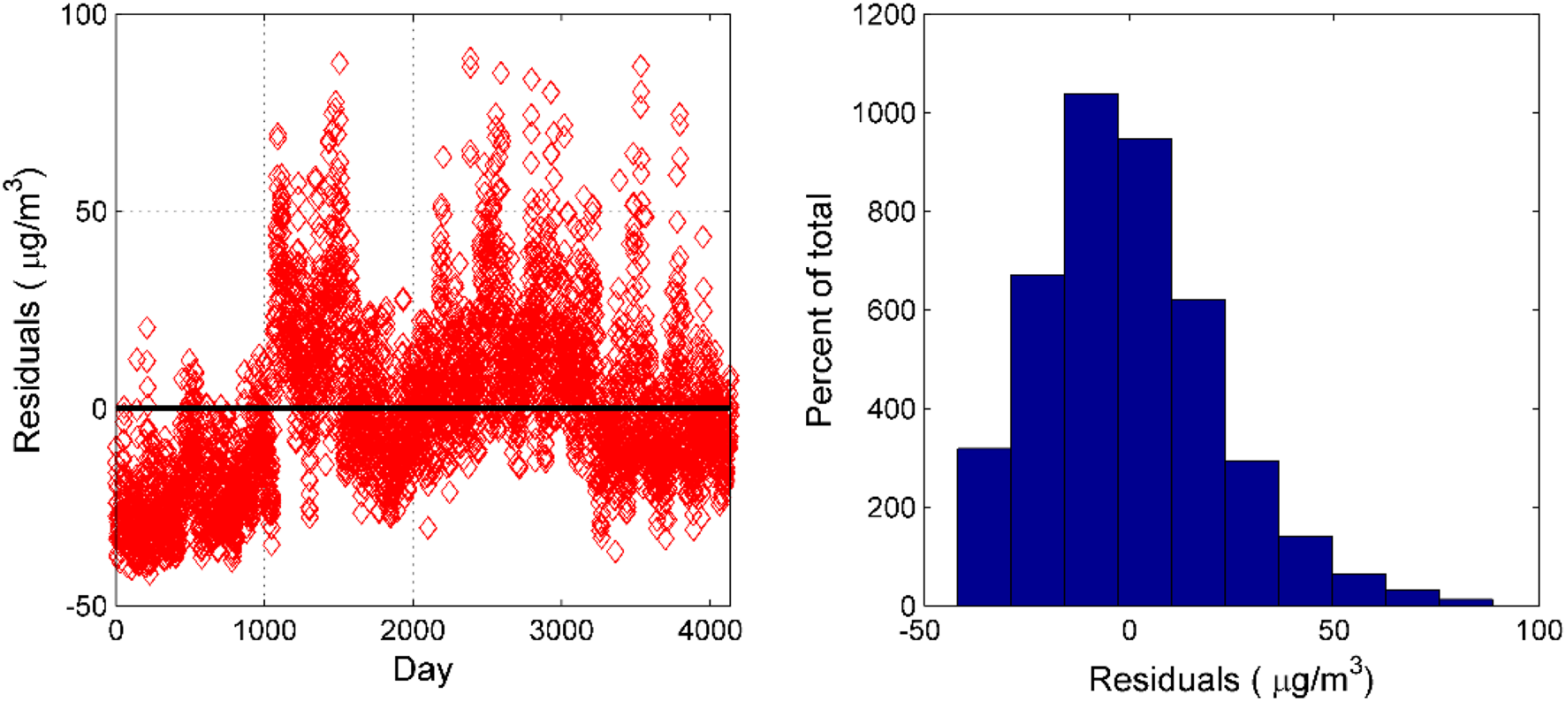
Residual test of PCR model. The residuals vs. day plot is seen on the left. The histogram of the residuals is seen on the right.

Since the cumulative contribution rate of the variance of the first six principal components exceeds 85%, extracting the first six principal components can better explain the information contained in the original variables. The principal component regression model with the concentration of NO_2_ at the national control point as the dependent variable and the 6 principal components extracted as independent variables is shown in Eq. (13), Where ${P}_{1},{P}_{2},\cdots ,{P}_{6}$ represent the first 6 principal components. The p-value of F test in the principal component regression model is less than 0.01, indicating that at a significant level of 0.01, the variables introduced into the model have a significant effect on the NO_2_ concentration as a whole. The p-values of the six principal component t-tests introduced into the model are all less than 0.01, indicating that at the significance level of 0.01, each independent variable introduced into the model has a significant effect on the NO_2_ concentration. The multiple correlation coefficient in the model is 0.471, indicating that the fitting effect needs to be improved. It can be seen from Fig. 6 that the error term basically meets the requirements, but the error value of some points is large, which affects the correction effect of the model on the measurement data of the miniature air quality detector.

13 $$y = 32.644 + 3.731P_{1} + 1.277P_{2} - 8.368P_{3} - 3.5P_{4} + 1.054P_{5} - 5.715P_{6}$$

### Construction of PCR–SVR model

Because the PCR model does not have a very good correction effect on the pollutant concentration, it shows that the influencing factors have a nonlinear effect on the pollutant concentration. We use the SVR model to improve the prediction effect. The SVR model is more sensitive to the choice of parameters and kernel functions. Therefore, the correct method to select the kernel function parameters and penalty coefficients is very important to the performance of the SVR and the calibration accuracy of the miniature air quality detector. This article uses the K-CV statistical analysis method to learn training samples, gradually changes the values of the SVR model parameters to obtain the best combination of parameters^19,32^, and then establishes a regression model for fitting.

In order to avoid the magnitude difference between the factors and eliminate the influence of each factor due to the different magnitudes and units, firstly, each predictor is normalized. Equation (14) is a normalized formula, where a, b are the maximum and minimum values of the original data c, and d, e are the mapping ranges, and the values here are 2 and 1.

14 $$y = \frac{{(y_{\max } - y_{\min } )(x - x_{\min } )}}{{x_{\max } - x_{\min } }} + y_{\min }$$

**Figure 7:**
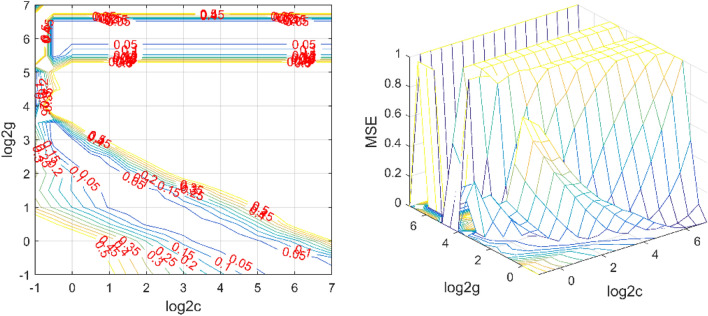
The K-CV statistical analysis method learns training samples. The SVR parameter selection result map (contour map) is seen on the left. The SVR parameter selection result graph (3D view) is seen on the right.

When building an air quality model, take the data measured at the self-built point and the predicted value of the PCR model as input, and the NO_2_ concentration at the national control point as output. According to the principle of approximately 7:3 for the training set and test set, 3000 sets of data were randomly selected from 4135 sets of data as the training set, and the other 1135 sets of data were used as the test set. The radial basis kernel function was used for SVR modeling, and the K-CV statistical analysis method was used to learn the training samples. After many times of cross-validation learning and training, a PCR–SVR regression model for NO_2_ concentration prediction was finally established. Figure 7 shows the process of K-CV statistical analysis method learning training samples, where the best c value is 2.8284, the best g value is 2.8284, and CVmse value is 0.005841.

**Figure 8:**
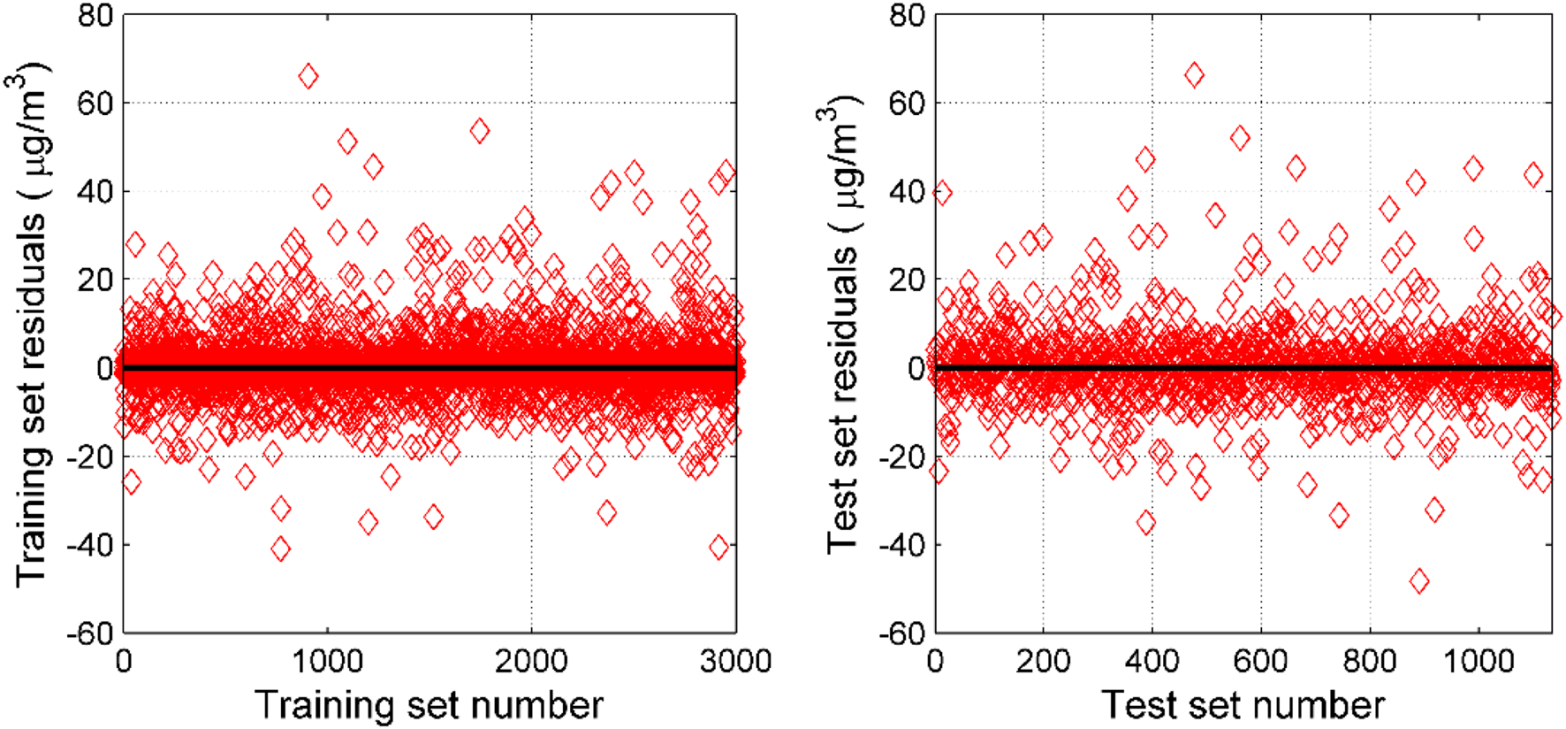
Residual test of PCR–SVR model. The training set residuals vs. training set number plot is seen on the left. The test set residuals vs. test set number plot is seen on the right.

It can be seen from Fig. 8 that the residuals of the training set and the test set have been greatly improved compared to the PCR model. The residual fluctuations of the training set and the test set are almost the same, indicating that the generalization ability of the model is very well. Using this model to correct the measurement data of the miniature air quality detector has a significant improvement.

### Construction of PCR–SVR–ARMA model

Although the prediction effect of NO_2_ concentration by the PCR–SVR model is good, a series of residual time series data are still obtained. The residual of some points in the model is still large (the maximum residual is 66.337 μg/m^3^). This paper uses the ARMA model to further mine the residual information to improve the prediction accuracy of the entire model.

**Figure 9:**
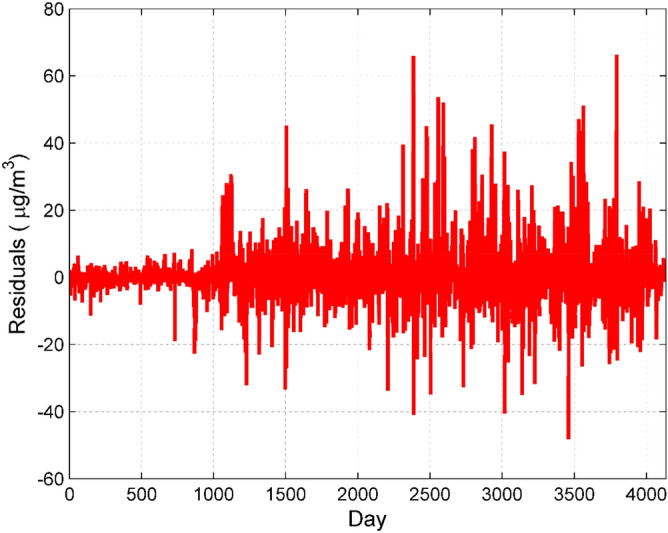
Stationary test of residual time series data of PCR–SVR model for prediction of NO_2_ concentration.

To construct the ARMA model of residual time series data, the stationarity test is first required. It can be seen from Fig. 9 that the residual fluctuates around a constant, which is a stationary series. Therefore, there is no need to differentiate the time series and make sure that d = 0. After determining the difference order d, we then determine the parameters p and q in the ARIMA model. The autocorrelation coefficient and partial autocorrelation coefficient of the time series can determine the value interval of the parameters p and q. Then compare Akaike Information Criterion (AIC) and Bayesian Information Criterion (BIC). According to the minimum information criterion, take the smaller value of AIC and BIC. With the help of SPSS20.0, the order of the ARMA model is finally determined p = 3, q = 8, and the residual time series data correction model is ARMA (3, 0, 8).

The white noise test was performed on the residual sequence, and the results showed that the Box-Ljung Q statistic difference was not statistically significant (P value greater than 0.05), and the model was significantly established^30,33^. Use the optimal ARMA model to predict the residual of the PCA-SVR model, and add the residual prediction result and the PCA-SVR prediction result to obtain the final prediction result of the NO_2_ concentration. The same method described above can be used in the prediction of the concentration of the other five types of pollutants, and the data of the miniature air quality detector can be corrected with the help of the predicted value.

## Discussion

**Figure 10:**
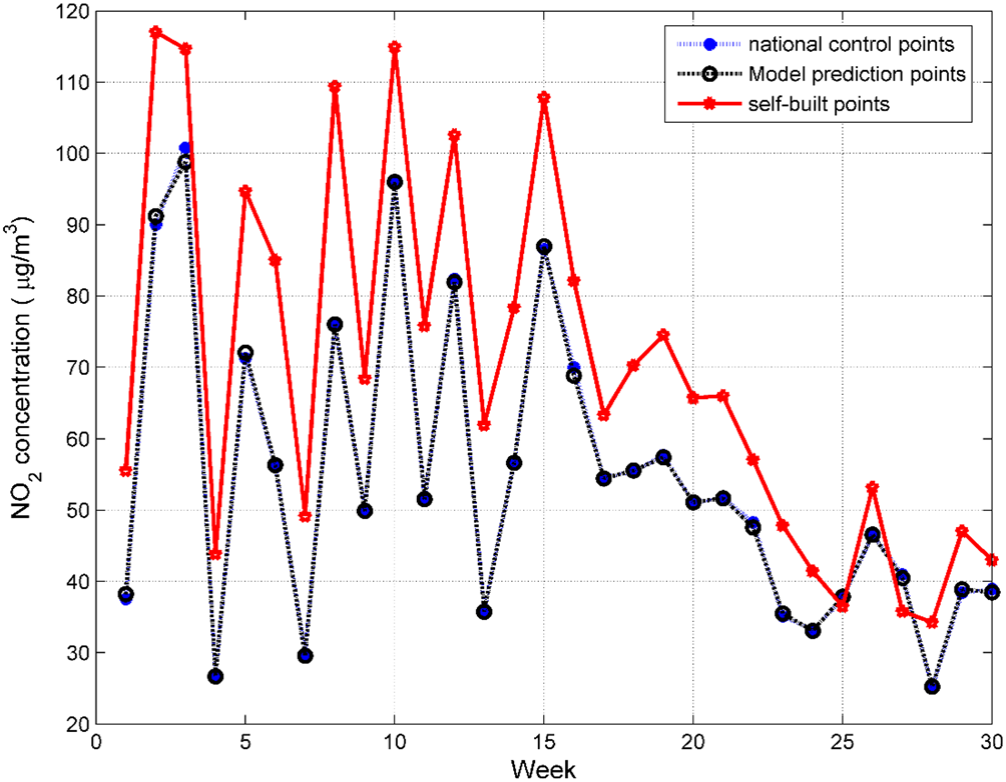
Comparison of the weekly average concentration of NO_2_ between national control points, PCR–SVR–ARMA model calibration points and self-built points.

Human activities have a significant effect on the concentration of pollutants in the air, and human activities are cyclical. We first averaged the concentration of NO_2_ in a one-week period, and then plotted the national control point data, PCR–SVR–ARMA model fitting value, and self-built point data into a line chart. In Fig. 10, the blue curve is the national control point data, the black curve is the model fitting value, and the red curve is the self-built point data. It can be seen that the model fitting data is very similar to the national control point data. On the contrary, the data of many self-built points are quite different from the national control point data. The PCR–SVR–ARMA model has a good correction effect on the self-built point data.

In the air quality prediction problem, PCR model, SVR model, PCR–SVR and PCR–SVR–ARMA model can all fit the pollutant concentration. In addition, artificial neural networks are one of the most commonly used methods to predict the concentration of air pollutants. At present, the multilayer perceptron (MLP) neural network is the most frequently used artificial neural network. We divide the sample into training set and test set at a ratio of 7:3, and use SPSS20.0 to find the optimal number of neurons to predict the concentration of six types of pollutants^16,34^. This article uses Root Mean Square Error (RMSE), Mean Absolute Error (MAE), and relative Mean Absolute Percent Error (MAPE) (Eqs. (14)–(16), where ${y}_{i}$ is the concentration of the six types of pollutants at the national control point, ${w}_{i}$ is the concentration after the self-built point is corrected) to evaluate all models^23^. The specific results are shown in Tables 4, 5 and 6.

**Table 4:** RMSE of six types of air pollutant concentrations between self-built points, model forecast values and national control point.

Input variable	Self-built points	PCR	SVR	PCR–SVR	PCR–SVR–ARMA	MLP
PM2.5	22.436	15.385	8.649	6.522	6.151	10.777
PM10	66.263	28.936	11.656	12.368	10.769	19.126
CO	0.679	0.362	0.175	0.169	0.139	0.304
NO_2_	37.183	21.474	7.725	7.612	6.893	13.216
SO_2_	26.24	15.757	4.116	4.098	3.915	9.984
O_3_	45.673	25.08	11.304	11.23	9.546	18.603

**Table 5:** MAE of six types of air pollutant concentrations between self-built points, model forecast values and national control point.

Input variable	Self-built points	PCR	SVR	PCR–SVR	PCR–SVR–ARMA	MLP
PM2.5	18.181	12.248	5.821	4.388	4.202	7.763
PM10	50.151	22.76	7.080	7.547	6.803	13.184
CO	0.549	0.283	0.110	0.105	0.088	0.237
NO_2_	29.838	16.918	4.658	4.597	4.275	9.991
SO_2_	12.867	10.792	2.116	2.103	2.003	7.246
O_3_	36.63	19.783	7.647	7.583	6.435	14.396

**Table 6:** MAPE of six types of air pollutant concentrations between self-built points, model forecast values and national control point.

Input variable	Self-built points	PCR	SVR	PCR–SVR	PCR–SVR–ARMA	MLP
PM2.5	0.447	0.335	0.133	0.108	0.105	0.185
PM10	0.887	0.478	0.107	0.114	0.105	0.210
CO	0.478	0.347	0.112	0.107	0.088	0.283
NO_2_	2.129	0.965	0.170	0.168	0.154	0.471
SO_2_	0.685	0.75	0.131	0.13	0.123	0.530
O_3_	4.322	1.399	0.373	0.368	0.295	1.002

15 $$RMSE=\sqrt{\frac{1}{n}\sum_{i=1}^{n}{({y}_{i}-{w}_{i})}^{2}}$$

16 $$MAE=\frac{1}{n}\sum_{i=1}^{n}\left|{y}_{i}-{w}_{i}\right|$$

17 $$MAPE=\frac{1}{n}\sum_{i=1}^{n}\left|\frac{{y}_{i}-{w}_{i}}{{y}_{i}}\right|$$

It can be seen that in addition to the MAPE of SO_2_, the errors of each model are improved compared with the errors of the self-built points. The RMSE, MAE, and MAPE of the PCR–SVR–ARMA model are the smallest among all models. Therefore, using the PCR–SVR–ARMA model to correct the self-built point data has the best effect. Comparison of the PCR–SVR–ARMA model of the six types of air pollutant concentrations with the self-built point data: the highest accuracy improvement rate of RSME is the SO_2_ concentration model, which has increased by 85.1%; the lowest accuracy improvement rate of RMSE is the PM2.5 concentration model, which has increased by 72.6%; the highest accuracy improvement rate of MAE is the PM10 concentration model, which has increased by 86.4%; the lowest accuracy improvement rate of MAE is the PM2.5 concentration model, which has increased by 76.9%; the highest accuracy improvement rate of MAPE is the O_3_ concentration model, which has increased by 93.2%; the lowest accuracy improvement rate of MAPE is the PM2.5 concentration model, which has increased by 76.5%. The larger the error between the national control point and the self-built point, the higher the accuracy improvement rate of the PCR–SVR–ARMA model, which indicates that the model has a very obvious correction effect on the self-built point data. The fitting values of the six types of air pollutant concentrations and the regression straight line slope of the national control point data are very close to 1, which also confirms the accuracy of the model.

In the issue of air quality prediction, the monitoring qualification rate is a matter of great concern. We stipulate that the pollutant forecast error is less than 20% as the forecast qualified, and the error forecast error exceeds 20% as the forecast unqualified. Table 7 shows the monitoring qualification rate of self-built points and each model. It can be seen that the PCR–SVR–ARMA model performs better than other models in predicting the qualified rate of various pollutant concentrations.

**Table 7:** Monitoring qualification rate of six types of air pollutant concentrations between self-built points, model forecast values and national control point.

Input variable	Self-built points	PCR	SVR	PCR–SVR	PCR–SVR–ARMA	MLP
PM2.5	0.304	0.465	0.804	0.861	0.864	0.710
PM10	0.205	0.393	0.847	0.833	0.860	0.638
CO	0.154	0.437	0.846	0.857	0.897	0.629
NO_2_	0.149	0.208	0.702	0.703	0.732	0.395
SO_2_	0.243	0.272	0.846	0.848	0.851	0.432
O_3_	0.205	0.291	0.600	0.607	0.656	0.425

## Conclusions

The air quality index (AQI) is a dimensionless index that quantitatively describes the condition of air quality. Many countries use AQI indicators to evaluate air quality. The main pollutants involved in air quality evaluation are PM2.5, PM10, CO, NO_2_, SO_2_, O_3_, etc. Therefore, to achieve air quality monitoring, real-time monitoring of the concentration of "two dust and four gases" is very important.

In order to monitor the concentration of various pollutants, many countries have established national control points. Although the national control point monitors pollutants more accurately, due to its high deployment control cost and high maintenance cost, it can only be deployed and controlled in developed cities or more important locations, and it is difficult to achieve full deployment control. The development of miniature air quality detectors has greatly alleviated this problem. However, since the built-in sensor is susceptible to interference from other irrelevant factors, the monitoring accuracy rate needs to be improved.

Aiming at the problem of data correction of the miniature air quality detector, we proposed a combined air quality prediction model^35,36^ based on principal component regression, support vector regression and autoregressive moving average model. The PCR–SVR–ARMA model was successfully applied in the calibration data of the miniature air quality detector.

The PCR–SVR–ARMA model we gave is very effective in predicting six types of pollutants. The data used in the model is 4135 groups, the time span is 206 days, and the data for all four seasons are involved, so the model is very stable. It can play a good role in correcting the self-built point data, and provide an important decision basis for the scientific arrangement of the miniature air quality detector. The climate conditions and pollutant concentrations in different regions are very different, which makes this model not necessarily applicable to different regions. The future direction of our research is to reasonably extend this model to other regions. We can also try to extend this model to other environmental monitoring problems.
